# Genome wide association studies and candidate gene mining for understanding the genetic basis of straw silica content in a set of *Oryza nivara* (Sharma et Shastry) accessions

**DOI:** 10.3389/fpls.2023.1174266

**Published:** 2023-05-30

**Authors:** Rakshith S. R. Gowda, Sandeep Sharma, Ranvir Singh Gill, Gurjit Singh Mangat, Dharminder Bhatia

**Affiliations:** ^1^ Department of Plant Breeding and Genetics, Punjab Agricultural University, Ludhiana, India; ^2^ Department of Soil Science, Punjab Agricultural University, Ludhiana, India

**Keywords:** *Oryza nivara*, rice, straw silica content, stubble burning, population structure, genome-wide association study, candidate genes

## Abstract

Rice is a high-silica (SiO_2_·nH_2_O) accumulator. Silicon (Si) is designated as a beneficial element associated with multiple positive effects on crops. However, the presence of high silica content is detrimental to rice straw management, hampering its use as animal feed and as raw material in multiple industries. Rice straw management is a serious concern in north-western India, and it is eventually burned *in situ* by farmers, contributing to air pollution. A practical solution could lie in reducing the silica content in rice while also attaining sound plant growth. A set of 258 *Oryza nivara* accessions along with 25 cultivated varieties of *Oryza sativa* was used to assess the variation in straw silica content using the molybdenum blue colorimetry method. A large continuous variation was observed for straw silica content in *O. nivara* accessions, ranging from 5.08% to 16%, while it varied from 6.18% to 15.81% in the cultivated varieties. The *O. nivara* accessions containing 43%–54% lower straw silica content than the currently prominent cultivated varieties in the region were identified. A set of 22,528 high-quality single nucleotide polymorphisms (SNPs) among 258 *O. nivara* accessions was used for estimating population structure and genome-wide association studies (GWAS). A weak population structure with 59% admixtures was identified among *O. nivara* accessions. Further, multi-locus GWAS revealed the presence of 14 marker-trait associations (MTAs) for straw silica content, with six of them co-localizing with previously reported quantitative trait loci (QTL). Twelve out of 14 MTAs showed statistically significant allelic differences. Thorough candidate gene analyses revealed the presence of promising candidate genes, including those encoding the ATP-binding cassette (ABC) transporter, Casparian thickening, multi-drug and toxin extrusion (MATE) protein, F-box, and MYB-transcription factors. Besides, ortho-QTLs among rice and maize genomes were identified, which could open ways for further genetic analysis of this trait. The findings of the study could aid in further understanding and characterizing genes for Si transport and regulation in the plant body. The donors carrying the alleles for lower straw silica content can be used in further marker-assisted breeding programs to develop rice varieties with lower silica content and higher yield potential.

## Introduction

Ever since the green revolution, rice production has tripled (FAOSTAT, 2021), keeping up with the rising population in the world. Proportionate to the increase in rice grain production, rice straw residue generation has also multiplied over the years, thus posing scientists all over the world with the perplexing query of rice straw management. In India, the north-western states of Punjab, Haryana, and parts of Uttar Pradesh produce large quantities of rice and, in turn, generate copious amounts of rice straw residue. Rice straw is notoriously difficult to manage as rice is a high silica accumulator. There are a myriad of alternative options proposed to handle rice straw, such as animal feed, bioethanol production, bedding material, and as raw material in multiple industries such as paper and pulp, pyrolysis, etc. Although some of these options even carry the potential for a significant income thrust for farmers, most of the suggested management practices are blatantly unpopular due to high investment in terms of labor and machinery for straw collection and transport (Singh et al., 2022). More importantly, the significant negative impact of the high silica content in rice straw is affecting the alternate usage of the same. In addition, an intensive agriculture system and a much shorter interlude between different crops lined up in a cropping system in these areas result in a significantly shorter period left available for straw management. The fact that most of the rice straw residue generated goes unutilized and is eventually burned *in situ* by the farmers is more of a compulsion than a choice to resort to the distressing practice of stubble burning. Stubble burning not only causes the emission of greenhouse gases on a large scale into the atmosphere, causing global warming, smog, and respiratory illnesses, but also wrecks soil health and microbiota (Agarwal et al., 2012; Lohan et al., 2018; Dutta et al., 2022). A large-scale survey of the farmers who indulge in the practice of stubble burning revealed that the farming community was entirely aware of the ill-effects of stubble burning but was compelled to resort to the action anyway owing to shortcomings in alternate straw management options (Bhattacharyya et al., 2021).

Silicon (Si) exists in the soil in the form of either minerals such as quartz, feldspar, silicon dioxide, aluminosilicates, and mica, or in liquid states as dissolved forms in soil solution, including silicic acid–inorganic compound complexes, monosilicic acid, and polysilicic acid (Schaller et al., 2021). Among these forms, monosilicic acid (H_4_SiO_4_) is the plant-available form of Si, which is taken up by the plants and later accumulated as hydrated silica (SiO_2_·nH_2_O). Plants exhibit differential abilities to take up and transport monosilicic acid, based on which they are partitioned into three categories: (i) high silica accumulators, which accumulate more than 1% silica content; (ii) intermediate accumulators, which accumulate 0.5%–1% silica content; and (iii) non-accumulators, which accumulate less than 0.5% silica content, on a dry weight basis (Jones and Handreck, 1967; Takahashi et al., 1990). Rice comes in the first category, accruing up to 18% silica in the plant body, most of which is found in polymerized form in the extracellular regions. Other cell wall components such as lignin, cellulose, and hemicellulose have been demonstrated to interact with silica present in plant bodies (Guerriero et al., 2016; Coskun et al., 2019).

The dynamics of silicon (monosilicic acid as the plant-available form of Si) transport and regulation in the plant body have been deciphered quite well, although there are significant gaps regarding understanding the exact mechanisms and coordinated functioning of the transporter genes. There have been a few studies in the past that have identified QTL and cloned genes responsible for Si uptake and transport, including *Lsi1* (low silicon gene 1) (Ma et al., 2006), *Lsi2* (low silicon gene 2) (Ma et al., 2007), *Lsi3* (low silicon gene 3) (Yamaji et al., 2015), and *Lsi6* (low silicon gene 6) (Yamaji et al., 2008). *Lsi1* belongs to the nodulin26-like protein subfamily of the aquaporin protein family and is primarily responsible for the passive influx of Si from the rhizospheric solution. Subsequently, the *Lsi2* gene, which is delimited to the plasma membrane of the endodermal cells of the roots, brings about the ATP-dependent transport of Si from the root cells to the apoplast (Ma et al., 2007; Ma et al., 2011). On the other hand, *Lsi6* is responsible for the differential distribution and deposition of Si across different shoot and leaf tissues (Yamaji et al., 2008). The spatial and temporal nuances and synchronies in the expression of these genes are responsible for the uptake and accumulation of Si from the soil. A few QTL mapping studies have also been conducted (Dai et al., 2005; Wu et al., 2006; Bryant et al., 2011; Pinson et al., 2022) to identify genomic regions associated with silica content in rice. All these studies have not encompassed the validated transporter genes *Lsi1*, *Lsi2*, *and Lsi6*, except Pinson et al. (2022), who have reported an association of *Lsi1* 2 MB away from the peak SNP *in* one of the QTLs. This also indicates the presence of a wide natural variation in the genes involved in Si transport and regulation present in rice germplasm. In addition, the Si transporters are also responsible for the uptake of analogues of Si, including arsenic (As) and germanium (Ge) (Ma and Yamaji, 2006; Rains et al., 2006; Nikolic et al., 2007; Ma et al., 2008; Delvigne et al., 2009; Li et al., 2009).

Si is deemed to be a beneficial element in plant nutrition and is associated with a plethora of positive effects such as biotic and abiotic stress resistance, improved quality, and quantity of produce, mechanical strength, and lodging resistance. Although the mechanism of how Si brings about beneficial effects in plants is contended, the fact that Si is detrimental to digestibility and thus management of straw is uncontended (Shewmaker et al., 1989; Mayland and Shewmaker, 2001; Coskun et al., 2019) and demands immediate attention to combat stubble burning. In addition, the industrial use of rice straw for bioethanol production, the paper and pulp industry, straw briquetting, biogas production, and pyrolysis is significantly hampered due to the high silica content in the straw (Parameswaran et al., 2010; Kurokochi and Sato, 2015). A pragmatic solution to the issue at hand would be to lower the silica content of rice varieties, striking a balance between sound plant growth and lower straw silica content, thus aiding in straw management. The current study sought the assistance of the wild annual progenitor species of cultivated rice, *O. nivara.* Wild species house tremendous allelic variation and have been utilized in the past for the breeding of many economically important traits (Khush and Ling, 1974; Tanksley and McCouch, 1997; Thanh et al., 2006; Kaladhar et al., 2008; Swamy et al., 2014; Haritha et al., 2018). It could be possible to identify donors in this rice germplasm that have a lower straw silica content, particularly in straw, in such a way that it does not hamper the plant’s growth and development. The study thus aimed to assess variation and identify promising QTL and candidate genes, thereby enriching the current understanding regarding the regulation of Si and related elements in plant bodies and identifying donors that could be used in future rice breeding programs.

## Materials and methods

### Plant material

The School of Agricultural Biotechnology, Punjab Agricultural University (PAU), Ludhiana, is maintaining many wild species accessions belonging to different genomes of rice, obtained from the International Rice Research Institute (IRRI), Philippines, and the National Rice Research Institute (NRRI), Cuttack, India. A set of 258 *O. nivara* accessions (**Table S1**) from this collection, along with 25 cultivated rice varieties belonging to *O. sativa* (**Table S2**), were utilized for the estimation of straw silica content. The 25 cultivated varieties included some of the most prominent varieties in the region, including both Basmati and non-Basmati types, the green revolution mega cultivar Taichung Native 1 (TN1), and a black aromatic cultivated variety (Chakhao Poireiton) of Eastern India. Five plants of each of the *O. nivara* accessions and 10 plants of each cultivated rice variety of *O. sativa* were sown during the *Kharif* season 2020–21 at the experimental fields, Department of Plant Breeding and Genetics, PAU, Ludhiana. The *O. nivara* accessions were sown at a plant × row spacing of 30 × 30 cm and cultivated rice varieties at 20 × 20 cm. All the recommended practices for rice cultivation were followed to raise a healthy crop (Anonymous, 2023).

### Straw silica estimation and statistical analysis

Straw samples consisting of all the above-ground parts except the panicles were collected at maturity from five plants in each accession of *O. nivara* and cultivated varieties. The samples included the stem and leaf parts, which are considered the straw, excluding the roots and the panicles of each genotype. The samples were chopped into smaller pieces. The chopped straw samples were placed in a hot air oven at a temperature of 70°C for a period of 7 days. The oven-dried samples were ground to a fine powder and sifted through a mesh sieve. The powder so obtained was again oven-dried at 60°C for 2 days. Further, the straw silica content from a 0.1 g powdered sample of each genotype was quantified using the autoclave-based digestion-mediated molybdenum blue colorimetry method given by Weimin et al. (2005) in two replicates. The distribution of mean straw silica content was plotted as a histogram, and the Shapiro–Wilk test was performed to check for a normal distribution. The statistical analysis to estimate significant differences in straw silica content among the *O. nivara* accessions and cultivated varieties was performed using the *lme4* (Bates et al., 2015) package, taking a completely randomized design (CRD).

### Genotype data analysis

All the *O. nivara* accessions were genotyped by sequencing (GBS) using the ddRADseq (double digest restriction-site associated DNA-seq) approach given by Peterson et al. (2012). Total genomic DNA was isolated using a CTAB (cetyl trimethyl ammonium bromide) extraction method (Murray and Thompson, 1980), with the chloroform-isoamyl alcohol purification step repeated twice to assure good-quality DNA. The ddRADseq-based GBS was outsourced to NGB (NextGen Bio) Diagnostics Pvt. Ltd., Noida, India. Data was received in the form of short sequence reads. Raw GBS data in the form of short reads of each line was aligned to *O. sativa* ssp. *japonica* cv. Nipponbare (IRGSP v1.0) as the reference genome using BWA (Burrows–Wheeler alignment) (Li and Durbin, 2009) with default parameters. SNPs were called using GATK (Genome Analysis Toolkit) (McKenna et al., 2010) and then filtered based on read depth ≥2, quality score >30, missing data <10%, and minor allele frequency (MAF) of 0.05. The final SNPs so obtained were further pruned to remove multiallelic SNPs using PLINK1.9 (Purcell et al., 2007). After the quality control process, a total of 22,528 informative SNPs were obtained and used for further analysis.

### Population structure and linkage disequilibrium analysis

The population structure among *O. nivara* accessions was determined using Structure v2.3.4 based on the Bayesian clustering algorithm (Pritchard et al., 2000). The input files for the program were prepared using PGDSpider software (Lischer and Excoffier, 2012). The K values were set from 1 to 10, and the admixture model had 100,000 replicates for burn-in periods and 100,000 replicates for MCMC (Markov Chain Monte Carlo) iterations. The best K was determined by Structure Harvester (Evanno et al., 2005; Earl and vonHoldt, 2012). Accessions with membership criterion values of ≥75% were assigned to a specific subpopulation. In addition, principal component analysis (PCA) was performed using the *prcomp* function as implemented in the GAPIT (Genome Association and Prediction Integrated Tool) package (Lipka et al., 2012) in R. Analysis of molecular variance (AMOVA) was performed as a measure of genetic diversity in the *poppr* package (Kamvar et al., 2014). The kinship matrix was derived from TASSEL v2.5 (Bradbury et al., 2007) and visualized using the GAPIT package.

Linkage disequilibrium (LD) decay analysis was performed by measuring the pairwise Pearson correlation coefficients of allele frequencies between the SNP markers using PopLDdecay (Zhang et al., 2019). The results were then plotted in R using a customized script.

### Multi-locus genome-wide association studies

MTAs were discerned using the ML-GWAS package mrMLM (multi-locus random SNP effect mixed linear model) in R (Zhang et al., 2020). The mrMLM integrates six different ML-GWAS models, namely, mrMLM, FASTmrMLM, FASTmrEMMA, pLARmEB, pKWmEB, and ISIS EM-BLASSO. Although the six models work generally on the same principle, they vary in their computational ability to identify significant MTAs and estimate the effects of QTNs (quantitative trait nucleotides) precisely. The significance of the MTAs was decided based on the logarithm of odds (LOD) value threshold of ≥3. The QTNs that were repeatedly identified for the trait by two or more of the six models were the reliable QTNs for the trait. The Kruskal–Wallis test for ascertaining allelic differences was performed and visualized in R.

### Candidate gene mining, expression analysis, and protein–protein interactions

The candidate genes in the QTL regions of the significantly associated SNPs were retrieved from the BioMart functionality of the EnsemblPlants website (http://plants.ensembl.org/index.html). The potential candidate genes were shortlisted based on scrupulous perusal of annotated functions, a literature review, gene ontology descriptions, and Interpro (https://www.ebi.ac.uk/interpro/) annotations. *In silico* expression analysis of the genes was conducted by harvesting the log2 transformed spatiotemporal expression data of six different tissues involved in Si metabolism, namely leaf blade, leaf sheath, roots, stem, lemma, and palea, from the RiceXPro database (https://ricexpro.dna.affrc.go.jp/GGEP/index.php). The data also consisted of different growth stages, viz., vegetative, reproductive, and ripening. The gene expression patterns were visualized using TBtools software (Chen et al., 2020). The STRING protein database (https://string-db.org/) was utilized to observe protein–protein interactions of the most probable candidate genes by applying a confidence level of 0.40 as the minimum interaction score. The interactions include evidence from text mining, experimental results, co-expression data, and a curated database.

### Identification of cis-regulatory elements

The potential binding sites of transcription factors having a role in the expression of candidate genes were determined to characterize the effect of cis-regulatory elements (CREs) putatively involved in Si dynamics in the rice plant body. For this purpose, the nucleotide sequence of 1,500 base pairs upstream of the transcription start site (ATG) of each of the most probable candidate genes was retrieved using the RSAT (Regulatory Sequence Analysis Tools) Plant Server (https://rsat.eead.csic.es/plants/RSAT_home.cgi) and uploaded into the PlantCARE database (https://bioinformatics.psb.ugent.be/webtools/plantcare/html/) in FASTA format. The regulatory elements identified only on the sense strand along with a matric value of 5 were retained for the analysis (Sharma et al., 2022).

### Identification of conserved genomic regions in different cereals

The candidate gene models from the QTLs identified in the study for straw silica content were checked for the presence of orthologues in the wheat and maize genomes. The gene sequences were retrieved, and BLAST (Basic Local Alignment Search Tool) was analyzed against wheat and maize genomes available in the EnsemblPlants database to discover conserved genomic regions among the cereals, and the orthologues were retrieved as genomic coordinates. The genes possessing a confidence in orthology score of 1 (high), along with a high percentage of identity with the wheat/maize genes,were accepted as orthologues.

## Results

### Straw silica content of *O. nivara* accessions and cultivated rice varieties

**Figure 1:**
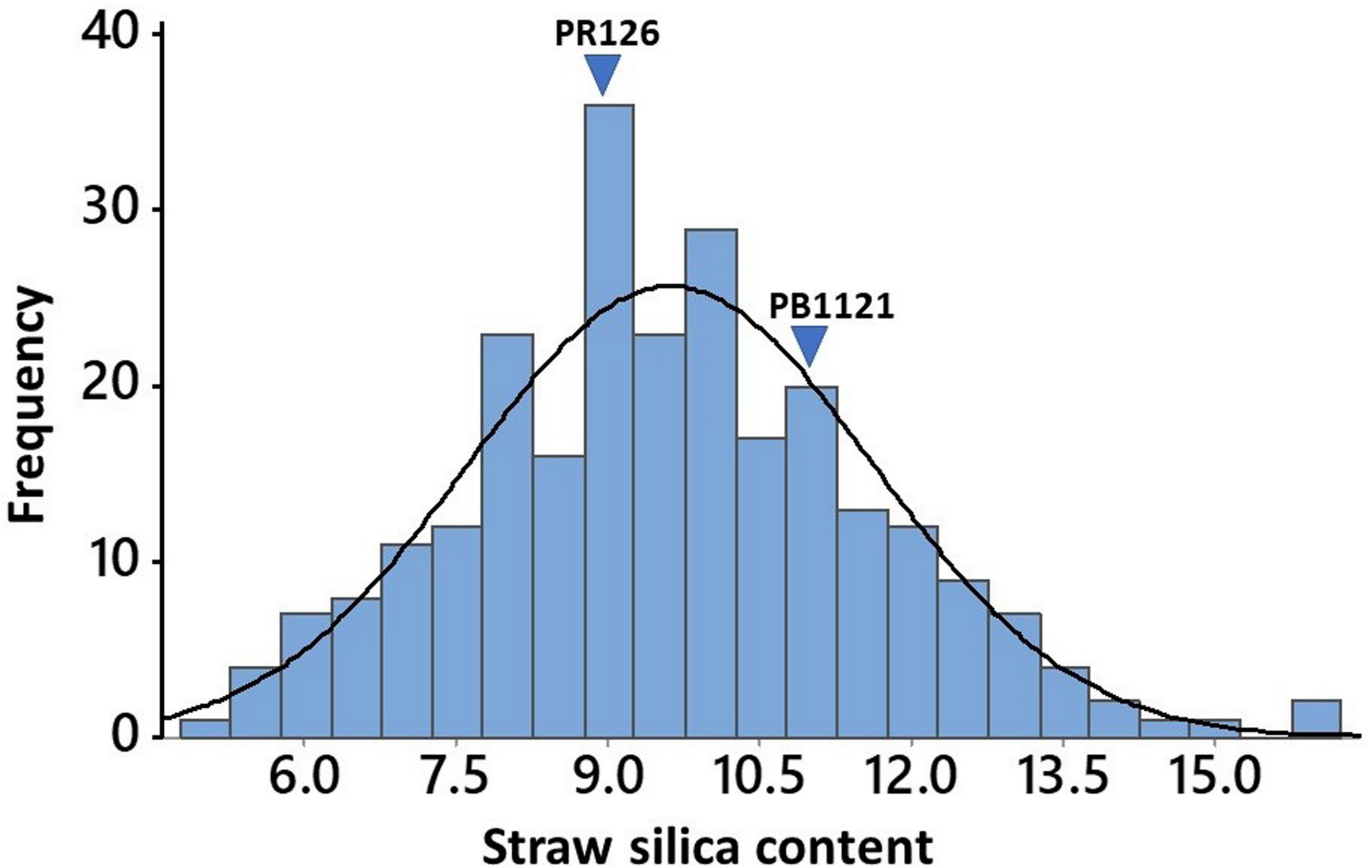
Frequency distribution of straw silica content in 258 *O. nivara* accessions, with arrows showing straw silica content of PR126 (*O. sativa*), a high yielding non-basmati cultivated variety, and Pusa Basmati 1121 (*O. sativa*), an elite Basmati cultivated variety of the region.

The 258 *O. nivara* accessions originally belonged to ten different countries, while most of the cultivated varieties used in the study include the prominent basmati and non-basmati varieties being cultivated in the Punjab state of India. Analysis of variance (ANOVA) indicated highly significant variation among the genotypes for straw silica content, while the replications were non-significant (**Table S3**). Among the *O. nivara* accessions, the straw silica content ranged from 5.08% to 16%, while it varied from 6.18% to 15.81% among the 25 cultivated varieties. The values were found to be normally distributed in the *O. nivara* accessions, as confirmed by the Shapiro–Wilk test for normality and as observed in the histogram (**Figure 1**). Among the cultivated varieties of Punjab, PR127 recorded the lowest (7.95%) straw silica content, while the highest (12%) was found in Pusa44 and PR106. There was no significant difference in straw silica content between basmati and non-basmati rice varieties. Evidently, *O. nivara* accessions containing 43%–54% lower straw silica content than the cultivated varieties were identified (**Table 1**). A total of 52 accessions were found to possess lower straw silica content as compared to the cultivated variety PR127, which recorded the lowest straw silica content among standard check varieties. Also, 94 accessions were found to contain lower straw silica content than PR126 (8.95%), a popular elite non-basmati variety, and 201 accessions were found to contain lower straw silica content than Pusa Basmati 1121 (11%), which is a popular elite basmati rice variety (**Table S2**). Interestingly, the cosmopolitan variety TN1 (Taichung Native-1) recorded the highest (15.81%) straw silica content.

**Table 1:** List of 10 best *O. nivara* accessions with lower straw silica content as compared to PR126 (*O. sativa*), an elite non-basmati variety, and PB1121 (*O. sativa*), an elite basmati rice variety.

Accession number	Country of origin	Si content (%)	% Decrease in Si w.r.t. PR126	% Decrease in Si w.r.t. Pusa Basmati 1121
IR106308	Cambodia	5.08	43.24**	53.81***
CR100184	India	5.52	38.32**	49.81**
CR100414	India	5.54	38.10**	49.63**
IR92759	Cambodia	5.54	38.10**	49.63**
IR105410	Sri Lanka	5.73	35.97**	47.90**
IR103841	Bangladesh	5.83	34.86**	47.00**
IR105806	Thailand	5.84	34.74**	46.90**
IR92799	Cambodia	5.90	34.07**	46.36**
CR100370	India	5.98	33.18**	45.63**
CR100418	India	6.08	32.06**	44.72**

**p-value significant at α = 0.001.

***p-value significant at α = 0.0001.

### Population structure in *O. nivara*

**Figure 2:**
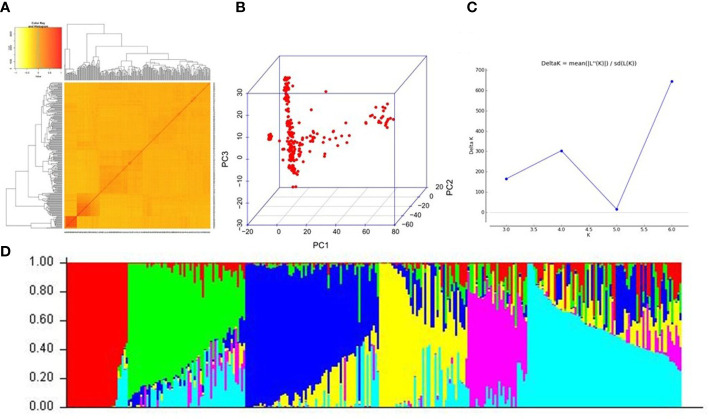
Population structure of the 258 *O. nivara* accessions based on 22,528 SNPs **(A)** Kinship heatmap depicting genetic distance between the accessions **(B)** Principal component analysis (PCA) plot indicating the clustering of the 258 *O. nivara* accessions into subpopulations **(C)** Magnitude of delta K values against a putative K range, indicating a peak at delta K = 6 **(D)** Population structure of 258 *O. nivara* accessions at K = 6.

After stringent filtering, we obtained a total of 22,528 high-quality SNPs distributed uniformly across the 12 chromosomes (**Figure S1**). The 22,528 SNPs spanned the entire genome of rice, with a frequency of one SNP per every 19 kb region. The genetic relationship among the accessions was assessed by generating the kinship matrix derived from the filtered SNPs. The majority of the pairwise kinship values were close to 0, and only 11.02% of the pairs scored pairwise kinship values >0.05, indicating that most of the accessions are distantly related to each other and cannot be clustered together into specific groups (**Figure 2A**). This provides preliminary indications of the presence of a weak population structure in the *O. nivara* accessions. However, principal component analysis (PCA) could reveal a distinction among the subpopulations (**Figure 2B**) that seems to divide the whole population into three subpopulations. But PCA’s division of *O. nivara* accessions into subpopulations could not provide information about admixtures. Further, population structure analysis using the Evanno method as implemented in Structure v2.3.4 software pointed out the presence of the highest ΔK peak at K = 6 (**Figure 2C**), indicating the presence of six subpopulations (**Figure 2D**). The membership probability criterion of 0.75 was the threshold for admitting an individual into a particular subpopulation, and individuals failing to cross the criterion threshold were considered admixtures. Out of the 258 individuals in the population, only 106 could be admitted into distinct subpopulations, while 152 were considered admixtures. Such high admixture levels in the population led to unclear distinctions between subpopulations indicating a higher degree of gene flow within the wild population and an accumulation of historical recombination events. Also, no correlation was observed between the geographical origin of the accessions and the subpopulation demarcation of the same. In addition, analysis of molecular variance (AMOVA) revealed that the variation among the subpopulations accounted for only 11.84% of the total variation, whereas the variation within the subpopulations accounted for 88.16% (**Table S5**). From these results, it can be asserted that there is a continuous variation and the absence of any strictly discrete categorization of the individuals into subpopulations. Based on PCA, Structure, and AMOVA, it was posited that the *O. nivara* population is weakly differentiated, consisting of 59% admixtures. Such a population type could be a decent choice for further GWAS.

### Multi-locus genome-wide association studies

**Figure 3:**
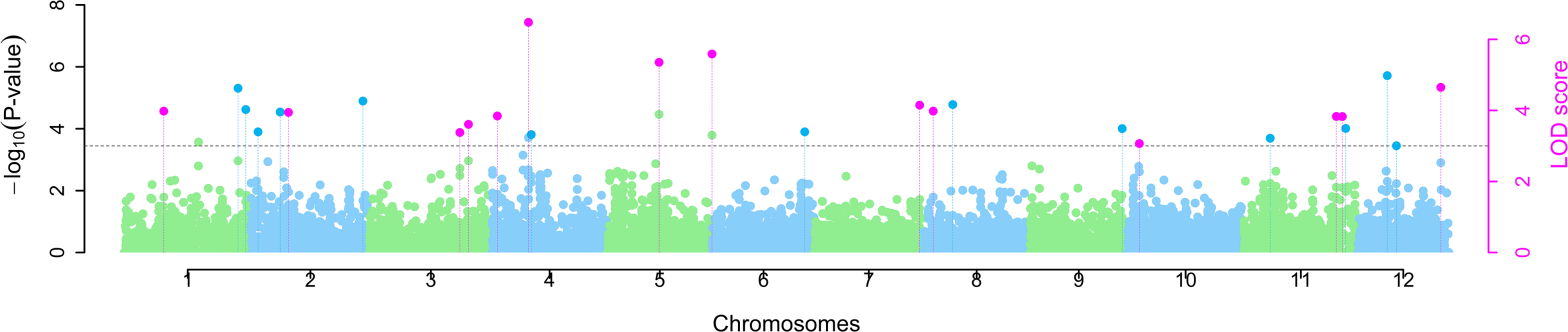
Manhattan plot depicting marker-trait associations (MTAs) for straw silica content among *O. nivara* accessions. Pink dots indicate significant MTAs identified simultaneously by two or more ML-GWAS models.

ML-GWAS implemented in mrMLM revealed 14 MTAs across 10 different chromosomes, except chromosomes 6 and 9, with LOD scores ranging from 3.01 to 7.23 (**Figure 3**, **Table 2**). These 14 MTAs were repeatedly identified as being significantly associated with the trait in two or more of the six ML-GWAS models. Two MTAs each were detected on chromosomes 3, 4, 5, and 11, while chromosomes 1, 2, 7, 8, 10, and 12 consisted of one MTA each. The most significant association occurred on chromosome 4, designated as *qSSi4.2*, which altered the straw silica content by 14.77%. A total of four out of the 14 MTAs, on chromosomes 3, 4, and 5, namely *qSSi3.2*, *qSSi4.2*, *qSSi5.1*, and *qSSi5.2*, respectively, were simultaneously found to be significant in five out of the six ML-GWAS models. Six MTAs identified in our study have been found to be co-localized with QTLs from other mapping studies for silica content (Dai et al., 2005; Wu et al., 2006; Pinson et al., 2022), thus providing analytical corroboration to our current study (**Table 2**).

**Table 2:** Marker Trait Associations (MTAs)/Single Nucleotide Polymorphisms (SNPs) associated with straw silica content in *O. nivara* accessions, along with previously reported QTLs for Silica, Arsenic (As) and Germanium (Ge).

QTL	SNP associated	Chrom	Position (bp)	LOD score	MAF^$^	Genotype	Models^#^	Previously reported QTLs colocalized
*qSSi1*	S1_12218963	1	12218963	3.89–4.07	0.49	AG	5, 6	
*qSSi2*	S2_10002377	2	10002377	3.12–4.43	0.48	GG	1, 2, 6	Talukdar et al. (2015) (Ge)
*qSSi3.1*	S3_25015087	3	25015087	3.31–3.44	0.22	TT	1,2	Wu et al. (2006) (Si)
*qSSi3.2*	S3_27398544	3	27398544	3.1–5.51	0.45	CC	1, 2, 4, 5, 6	Wu et al. (2006) (Si)
*qSSi4.1*	S4_1274437	4	1274437	3.6–4.06	0.29	TT	4, 6	
*qSSi4.2*	S4_9839223	4	9839223	4.22–6.67	0.15	GG	1, 2, 4, 5, 6	Pinson et al. (2022) (Si), Pinson et al. (2022) (As)
*qSSi5.1*	S5_13182293	5	13182293	4.2–6.3	0.49	GG	1, 2, 4, 5, 6	Dai et al. (2005) (Si)
*qSSi5.2*	S5_29790457	5	29790457	3.5–7.2	0.47	TT	1, 2, 4, 5, 6	
*qSSi7*	S7_27857697	7	2787697	4.05–5.76	0.42	GG	1, 2	Wu et al. (2006) (Si), Pinson et al. (2022) (Si)
*qSSi8*	S8_4062103	8	4062103	3.89–4.11	0.48	GG	5, 6	
*qSSi10*	S10_1674921	10	1674921	3.01–3.11	0.32	TT	5, 6	Wu et al. (2006) (Si)
*qSSi11.1*	S11_25225732	11	25225732	3.38–4.28	0.48	GG	1, 6	Pinson et al. (2022) (As), Liu et al. (2019) (As)
*qSSi11.2*	S11_25832818	11	25832818	3.79–3.86	0.38	AA	2, 5	Pinson et al. (2022) (As), Talukdar et al. (2015) (Ge)
*qSSi12*	S12_24560066	12	24560066	4.00–5.30	0.35	TT	1, 6	

^$^MAF, Minor Allele Frequency.

^#^1, mrMLM; 2, FASTmrMLM; 3, FASTmrEMMA; 4, pKWmEB; 5, pLARmEB, 6, ISIS EM-BLASSO.

### Estimation of allelic effects

**Figure 4:**
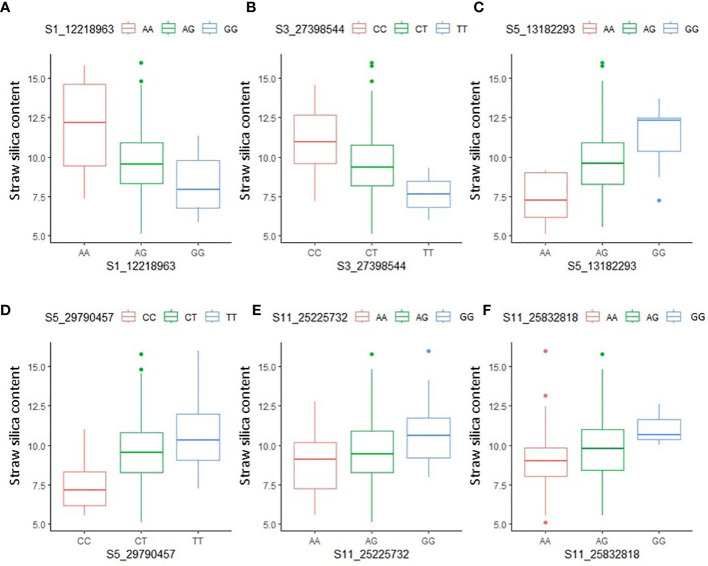
Boxplots depicting allelic effect differences of significant MTAs **(A)** S1_12218963 **(B)** S3_27398544 **(C)** S5_13182293 **(D)** S5_29790457 **(E)** S11_25225732 **(F)** S11_25832818, identified using ML-GWAS

The allelic effects of the significant MTAs were examined using the Kruskal–Wallis test. The chi-square values and probability (p) values indicated the presence of a significant statistical difference in allelic effects for 12 out of the 14 MTAs (**Table S6**). For the MTA S5_13182993, representing *qSSi5.*1, the accessions with allele ‘A’ had, on average, 3.96% lower straw silica content than the accessions containing the ‘G’ allele. Similarly, for the MTA S1_12218963, representing *qSSi1*, the accessions with the allele ‘G’ had, on average, 3.61% less straw silica content than those containing the ‘A’ allele (**Figure 4**). Interestingly, heterozygotes usually scored a straw silica value roughly around the midpoint between the two homozygotes in most of the MTAs.

### Candidate gene mining in QTL regions

A QTL region with significant MTAs was identified based on LD decay analysis. LD decay in the population was 150 kb, where LD dropped below 0.2 (**Figure S2**). The 300 kb region surrounding the significant MTAs was analyzed for the identification of candidate genes responsible for straw silica content. A total of 344 gene models were found in the 14 QTL regions of significant MTAs. The list was further filtered to include only those genes that were components of membranes and/or involved in transmembrane transport of Si, other nutrients, metals, and metalloids, along with other subsidiary genes involved in Si metabolism in plants. Overall, based on a scrupulous literature review, functional annotation description, gene ontology, and Interpro annotations, 57 genes were shortlisted, including those coding for transmembrane transporters, integral membrane components, Casparian thickenings, multi-drug and toxin extrusion (MATE) protein, F-box domains, MYB (myeloblastosis)-transcription factors, BTB (Broad-Complex, Tramtrack, and Bric a Brac) domain-containing protein, and SNARE (soluble *N-*ethylmaleimide-sensitive factor attachment protein receptors). In addition, 108 domains of unknown functions and hypothetical genes were also shortlisted. These were especially considered for downstream analysis because of the dearth of ample functional genomics-assisted studies of Si biology in rice. The shortlisted 165 genes, including the hypothetical genes, were rigorously examined for differential expression in the concerned tissues involved in Si uptake, distribution, and accumulation, viz., roots, stem, leaf sheath, leaf blade, lemma, and palea. The expression profiles of the candidate genes were stringently matched with those of the validated Si transporter genes as well as the subsidiary genes.

**Figure 5:**
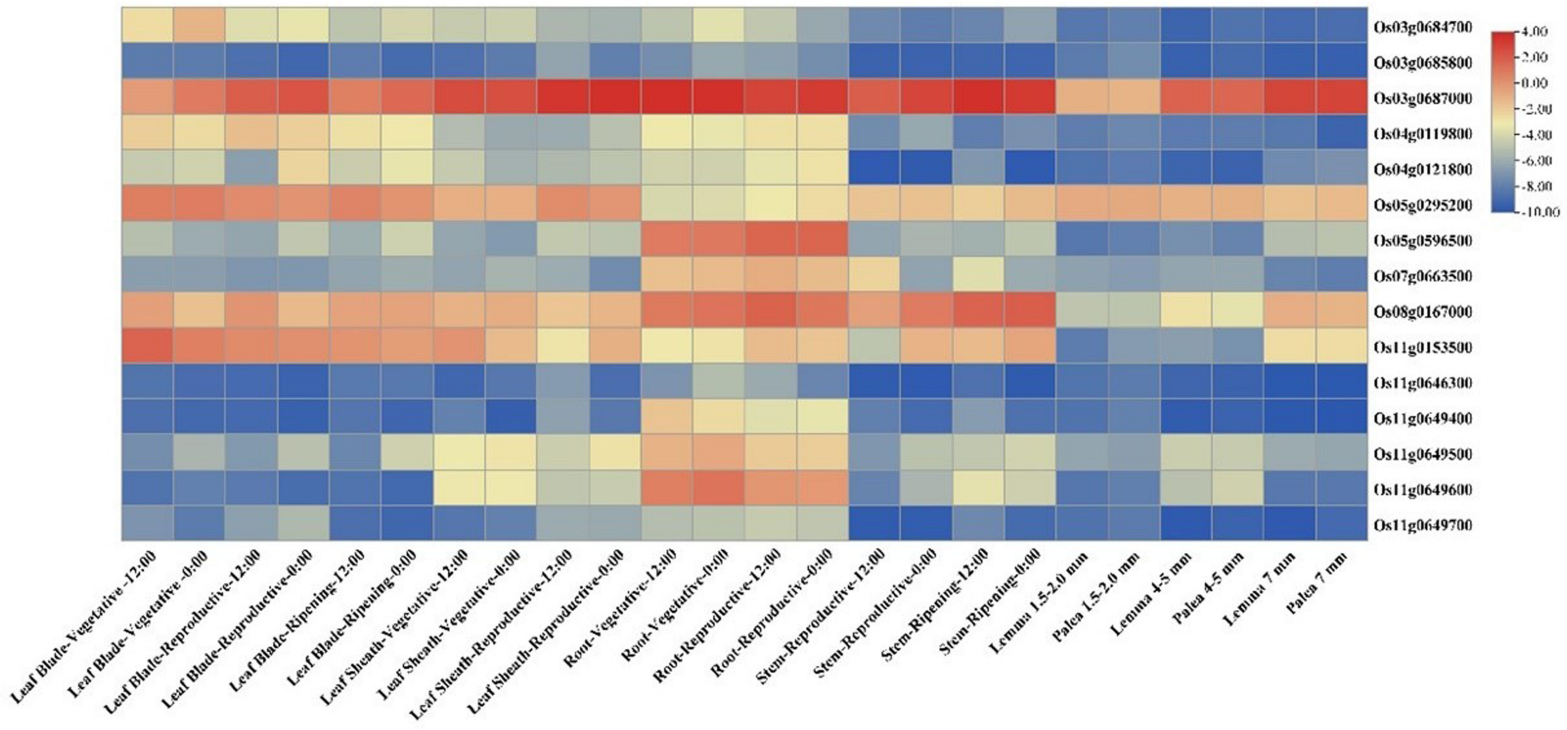
Expression heatmap depicting tissue-specific expression profiles of candidate genes (identified based on ML-GWAS among *O. nivara* accessions) having a role in silicon transport and regulation.

*In silico* expression analysis revealed 16 genes to be differentially expressed in the tissues, matching the expression profiles of the known Si transporter genes (**Figure 5**). Among the 16 genes, there were two transmembrane transporter genes in *qSSi8 and qSSi3.2*, respectively, i.e., *Os08g0167000*, which encodes ABC transmembrane transporter domain protein, and *Os03g0687000* present in *qSSi3.2*, which putatively encodes a nitrate transporter and belongs to the protein oligopeptide transport family. These genes are primarily expressed in the roots and stems of plants. A gene cluster was found in *qSSi11.2* consisting of four genes encoding Casparian strip membrane domains, responsible for Casparian strip formation in the endodermis of the roots. Another gene coding for the MATE (multi-drug and toxin extrusion) domain-containing protein, *Os02g0273800*, was found in the *qSSi2* region. Intriguingly, one non-protein-coding transcript was found through differential expression analysis, namely *Os04g0121800*, as a part of the *qSSi4.1* region. Other than the two hypothetical genes that lack functional annotation, all the other genes could be tied up with transport, metabolism, and gene regulation functions regarding Si in rice. The F-box and MYB-domains were also found across different QTLs responsible for the regulation of silica content (**Table S7**).

### Promoter analysis of putative candidate genes

**Figure 6:**
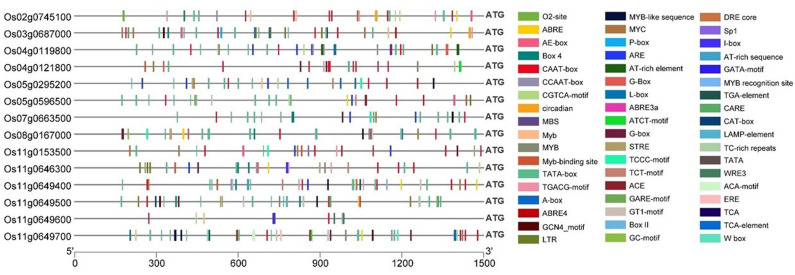
Cis-regulatory elements (CREs) in the promoter regions of *Lsi1* (*Os02g0745100*) and candidate genes (identified based on ML-GWAS among *O. nivara* accessions) having a role in silicon transport and regulation.

Cis-regulatory elements (CREs) in the promoter regions are instrumental in deciding and influencing gene regulation. Hence, promoter analysis was conducted for the candidate genes to identify the CREs in the promoter regions, i.e., 1.5 kb upstream of the 5’ end of the transcriptional start site (ATG) of the 13 differentially expressed genes. Among the 13 genes, a total of 381 CREs were discovered (**Figure 6**), out of which the common core CREs non-inclusive of any specific function, including TATA box and CAAT box regulators, accounted for a total of 182 CREs. The remaining 199 CREs could be grouped into plant growth and development, phytohormone response, and stress response. Unfortunately, there is a considerable dearth of literature regarding the function of CREs in Si transport and metabolism regulation; therefore, conclusive, or convincing outcomes could not be expected from promoter analysis. For partial redressal of the issue, the Si transporter gene *Lsi1* was also included in the analysis to ascertain the types and number of CREs it possessed. *Lsi1* consisted of four MYB-binding sites. MYB-domains are transcription factors that have previously been reported to be involved in Si regulation (Lu et al., 2002; Li et al., 2015; Roy, 2016; Chen et al., 2017; Wang et al., 2017; Xu et al., 2018; Chen et al., 2021). In addition, it contains the ABRE element, which is involved in the abscisic acid response and was reported to regulate the expression of the Si transporter genes (Lu et al., 2002; Chen et al., 2017; Chen et al., 2021). There was at least one and up to five MYB elements present in the promoter sequences of all the other genes. Also, the TC-rich repeats were related to defense and stress response, and CAT-box elements were found to be related to root meristem expression. The P-box and GARE-motifs were responsible for gibberellin response, and Me-JA and TGA-elements were responsible for jasmonate and auxin response, respectively. It can be concluded that MYB elements could be taken up for further studies, along with a thorough examination of other CREs that could be involved in Si and As regulation in the plant body. It can also be stated that the gene functioning mechanisms of the Si-transporters and all the regulatory and subsidiary genes involved with Si and As metabolism have a very complex functioning system, engaging in cross-talks with the regulatory elements, hormones, non-coding RNAs, external environmental conditions, and possibly epigenetic mechanisms governing the same.

### Conserved genomic regions associated with straw silica content in maize

**Figure 7:**
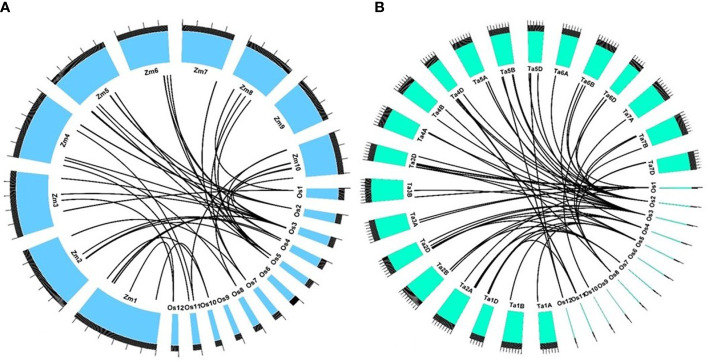
Syntenic relationship of genes present in QTL regions governing straw silica content in *O. nivara* accessions with **(A)** maize and **(B)** wheat. The physical sizes of the chromosomes are indicated by the ruler drawn above them, with larger and smaller tick marks at every 100 and 20 MB region, respectively.

All 344 gene models found in the QTLs identified in our study were BLAST-analyzed against the maize and wheat genomes. In maize, out of 344 genes, 104 were found to possess an orthology score of 1, showing at least 70% identity with the target gene. These genes were used to discern collinear genomic regions between rice and maize. In a particular QTL, if more than 60% of the genes possessing high-confidence orthology were found to agree with synteny and collinearity, they were reported as syntenic and collinear across the species. There were four significantly collinear regions, where the QTLs *qSSi1*, *qSSi2*, *qSSi7.1*, *and qSSi8* correspond to maize genomic regions 58.5–59.44 MB on chromosome 3, 136.5–138.58 MB on chromosome 5, 180.06–180.83 MB on chromosome 7, and 73.83–76.03 MB on chromosome 10, respectively (**Figure 7A**). It is to be noted that other than the orthologues of rice Si transporters present in maize (*ZmLsi1*, *ZmLsi2*, *and ZmLsi6*) (Mitani et al., 2009), there is a lack of available literature regarding silica transport and regulation. Hence, the syntenic regions reported in our study could be studied further in maize in the future. A similar attempt was made to the wheat genome as well. However, no significant syntenic regions were found across a single chromosome (**Figure 7B**).

## Discussion

### Straw silica content variation

The presence of silica in rice is associated with multiple biotic and abiotic stress regulation mechanisms. However, its high percentage in rice straw is detrimental to its management and alternate uses. Thus, farmers must resort to burning rice straw in the field, causing environmental pollution. A breeding solution to the issue would be to lower the silica content of rice varieties, keeping a balance with sound plant growth. Understanding the variation in straw silica content in rice germplasm, particularly wild relatives of rice, has not been attempted before, and this study explored this variation in the collection of diverse *O. nivara* accessions, a wild progenitor species of *O. sativa*, with the objective of identifying accessions that contain lower silica content.

Wild species contain enormous amounts of genetic diversity and have been used as donors of favorable alleles for improvement in multiple traits. *O. nivara* belongs to the ‘AA’ genome, similar to *O. sativa*, *and* both constitute the primary gene pool of rice along with other species. There is high compatibility and crossability between *O. sativa* and *O. nivara* (Oka, 1988; Brar and Khush, 2018), leading to the development of fertile hybrids under natural and artificial hybridization. Hence, *O. nivara* is an excellent donor to incorporate desirable variation into elite breeding rice lines. The study has identified promising donor accessions in *O. nivara*, IR106308, CR100184, CR100414, and IR92759, containing up to 43%–54% less straw silica content than the currently popular cultivated varieties of the region. Also, no significant difference was observed in the straw silica content between basmati and non-basmati rice varieties. It was intriguing that the cosmopolitan variety, TN1 (Taichung Native-1), which played a pivotal role in the green revolution, recorded a considerably higher silica content of 15.81% in the study. In addition, TN1 is used worldwide as a susceptible check in pest and pathogen resistance studies (Li et al., 2019), including rice brown planthopper (*Nilaparvata lugens*) (Tan et al., 2004; Deen et al., 2017), white-backed planthopper (*Sogatella furcifera*) (Wu and Khush, 1985; Yang et al., 2014), green leafhopper (*Nephotettix virescens*) (Vu et al., 2014), bacterial blight (*Xanthomonas oryzae*) (Kumar et al., 2012; Yugander et al., 2018), and rice stem borer (*Scirpophaga incertulas*) (Liu et al., 2018). The study thus raises the question of the evolutionary role of the presence of silica in rice. It is also noteworthy that studying the genotypic differences in straw silica uptake under soil Si homeostasis and its correlation with biotic stresses is different from studying the disease reaction after externally supplementing silica on one genotype, as reported in most of the previous studies. However, additional studies are required in this regard to dissect the role of silica in biotic and abiotic stresses.

### Population structure and genome-wide association studies in *O. nivara* accessions

Population structure assessment through different methodologies, including PCA, Structure, and AMOVA, depicted unclear differentiation among the *O. nivara* accessions, with 59% admixtures. A similar study in the *Oryza rufipogon* (a close relative of *O. nivara*) panel indicated slightly higher genetic diversity than the *O. nivara* panel used in our study (Kuroda et al., 2007; Li et al., 2020; Malik et al., 2022), indicating a higher rate of outcrossing and gene flow in *O. rufipogon* as compared to *O. nivara. O. nivara*, the annual form of *O. rufipogon* species complex (*ORSC*) and the proposed progenitor of the *indica* subpopulation of rice, is propounded to have originated more than once independently from different subpopulations of *O. rufipogon* (Sharma, 1965; Chang, 1976; Oka, 1988; Sharma, 2000; Cheng et al., 2003; Haritha et al., 2018; Eizenga et al., 2022). There is a considerable dearth of previous literature regarding extensive population genetic analyses in *O. nivara* populations. While the current panel was divided into six subpopulations using Structure, the clarity of differentiation was too low to call them distinct. Similar results were obtained from AMOVA and the kinship matrix. This indicates high gene flow and the accrual of historical recombination over thousands of years, resulting in higher genetic diversity, even though the outcrossing percentage of *O. nivara* is lower as compared to *O. rufipogon* (Kuroda et al., 2007; Li et al., 2020).

Single-locus GWAS models have been extensively used in the past for GWAS but suffer from caveats such as a high false-positive discovery rate and the requirement for corrections for multiple testing (Kaler and Purcell, 2019), leading to the use of the Bonferroni correction factor. It has been argued that the Bonferroni factor is highly stringent and results in the rejection of what would be true-positive associations, on account of its stringency (Su et al., 2018). Multi-locus GWAS models, on the other hand, address these issues due to the non-necessity of multiple corrections and true estimates of QTN effects. ML-GWAS in *O. nivara* accessions for straw silica content identified 14 MTAs, of which six were found to be co-localized with QTLs identified in previous mapping studies. Eight novel QTLs for straw silica content floating in *O. nivara* accessions have been identified in the current study.

### Relationship between silicon and arsenic

Arsenic (As) is a carcinogenic element that accumulates in the grains of rice. The non-existence of any threshold below which As does not cause cancer and related ailments in humans makes it more alarming. In rice, there has been an increase in As accumulation in the recent past as compared to previous years (Meharg, 2004). The dynamics of the Si–As relationship have been studied previously. The As uptake and accumulation in the *lsi1* mutant (low silicon mutant) line, which was originally used to identify the Si transporter gene *Lsi1*, were 71% and 53% lower in shoots and roots, respectively, as compared to the wild type (Ma et al., 2008). Zhang et al. (2008) reported that among two genotypes, CJ06 and TN1, the grain As content of TN1 was 2.44 times that of CJ06. Incidentally, the Si content of the TN1 variety was also found to be considerably higher at 15.81%, as found in our current study. In light of these facts, it can be hypothesized that the inherent properties of the transporter proteins that are coded by different alleles of the Si transporter genes also correspond in the same direction to As uptake and accumulation. This could potentially create a double-win situation, combating stubble burning and grain As at once, and further studies directed in this arena could manifest the relationship between As and Si.

Further, our study found three QTLs to be co-localizing with previously reported QTLs for As content. *qSSi4.2* was found to be co-localized with *qAs4*, while *qSSi11.1 and qSSi11.2* were co-localized with *qAs11-2* identified by Pinson et al. (2022). In addition, *qSSi11.1* was found to be co-localized with *qGAs18 and qGAs22* identified by Liu et al. (2019) (**Table 2**).

### Relationship between silicon and germanium

Along with As, Si is also analogous to germanium (Ge), due to which it is considered that it is transported by the same transporter genes as Si (Ma and Yamaji, 2006; Rains et al., 2006; Nikolic et al., 2007; Delvigne et al., 2009). However, in contrast to Si, Ge is highly phytotoxic and produces toxicity symptoms at even extremely low concentrations (above 1 μg/L) in crops like rice and horsetail, both of which are known to be high Si accumulators (Takahashi et al., 1976). It causes symptoms of distress, including necrosis, increased activity of peroxidases in barley, and characteristic brown spots on leaves in rice (Halperin et al., 1995). Ge was used as a proxy for tracing the amount of Si taken up by Ma et al. (2002), where rice mutants defective for Si uptake were screened using Ge kinetics. Plants with higher concentrations of Si were characterized by higher concentrations of Ge (Ma et al., 2001). Nikolic et al. (2007) reported that the processes of uptake into the roots and root-to-shoot transmission of both Si and Ge were extremely similar. The silicon efflux transporter gene *Lsi2* was identified based on Ge uptake and its correlation with silicon uptake. A set of mutant rice was screened by treating them with Ge and observing the manifestation of the deleterious symptoms. The mutants that did not display such deleterious effects were found to accumulate very low Si contents (Ma et al., 2007).

Ge is a metalloid and a semiconductor used in the electronic industries, gradually overthrowing the usage of Si (Lin et al., 2005). Since the usage of Ge has gained pace of late, the discharge of electronic waste has gone up and will continue to scale up in the coming years. Even though, as of today, the soils are not characterized as being contaminated with Ge, the days are not far away when such a predicament presents itself and stirs up the risks of potentially disrupting ecosystem equilibrium. Si content could be a reliable marker for Ge uptake and thus coincidentally assist in breeding lines with a lower ability to take up Ge, Si, and As, resulting in a triple win. This could be the way to look at it to accommodate breeding for climate change and climate-smart agriculture. Also, two QTLs, *qSSi2* and *qSS11.2*, identified in our study were found to be co-localized with *Ge5 and Ge6* QTLs, respectively, identified by Talukdar et al. (2015) for Ge content in rice (**Table 2**).

### Candidate genes for straw silica content

Based on *in silico* expression analysis, gene ontology searches, and a thorough literature review, promising candidate genes in the QTL regions involved in Si transport and regulation were identified. These could be novel genes existing in the natural population rather than the mutant genes *Lsi1*, *Lsi2*, *Lsi3*, *and Lsi6*.

*qSSi8* harbors an ABC transporter domain-containing transmembrane transporter protein, *Os08g0167000*. This gene was found to be expressed primarily in the roots and stem, followed by the lemma and palea. This follows the expected flow path of Si and As transport and distribution and is also backed by the fact that both elements are transported by the same transporters (Li et al., 2009). ABC transporters have been demonstrated to perform functions such as Si-induced formation of Casparian strips in the endodermis of the roots (*OsABCG25*) (Hinrichs et al., 2017) and reduced As accumulation in the rice grains by detoxification and vacuolar sequestration (*OsABCC1*) (Song et al., 2014). Hence, this gene most certainly warrants further studies with respect to Si and As uptake and metabolism in the plant body.

*qSSi11.*2 consists of a cluster of four genes, namely *Os11g0649400*, *Os11g0649500*, *Os11g0649600*, *and Os11g0649700*, belonging to the plant transmembrane domain and responsible for Casparian band formation (Roppolo et al., 2011). Casparian strips are significantly influential in the transport of nutrients and minerals by providing a stringent apoplastic diffusion barrier, preventing apoplastic bypass of nutrients and backflow of nutrients, ions, and water (Enstone et al., 2002; Geldner, 2013; Sakurai et al., 2015). Based on the simulative model as presented by Sakurai et al. (2015), when the Casparian strips are present, Si accumulated in the shoots is significantly higher in high Si-accumulators such as rice. In turn, the roots lacking the Casparian strips transport considerably fewer amounts of Si to the shoots (Sakurai et al., 2015). These genes could be studied further in depth to weave a stronger link between Si transport and root characteristics of the plant, an area of study that is still unclear.

*qSSi2* harbors a gene encoding a multi-antimicrobial extrusion protein (multidrug and toxic compound extrusion protein, MATE), *Os02g0273800*. One gene coding for the MATE protein, *OsMATE2*, was reported to modulate As transport. Upon constitutive expression, the gene decreased root-to-shoot transport of As in transgenic tobacco and reduced the accumulation of As in the grains in transgenic rice. In As-stressed conditions, the gene was found to be overexpressed six-fold, leading to a significantly increased accumulation of As in the grains (Das et al., 2018). Since As and Si are commonly transported by different transporters, the candidate gene in our study warrants thorough scrutiny, including studies about its role in Si and As transport and accumulation.

Intriguingly, one of the hypothetical proteins, *Os05g0295200*, present in *qSSi5.1* and shortlisted based on stringent expression in roots and stems, revealed that it interacts with a myriad of genes, two of them being NIP1-1 and NIP1-4. NIPs (Nodulin26-like intrinsic proteins) are the aquaporin family proteins responsible for water and solute transport across membranes (**Figure S3**). NIP1-1 is a putative arsenate transporter, and NIP1-4 is an aquaporin specific to arbuscular mycorrhiza (Sakurai et al., 2005), both of which are involved in Si transport. Since this hypothetical gene is primarily expressed in leaf blades and stems, and carries interactions with aquaporins, its inherent nature and the nature of interactions with aquaporins could be studied in detail.

*qSSi3.2* harbors an interesting gene, *Os03g0687000*, which is a transmembrane transporter gene functioning as a pH-dependent low-affinity nitrate transporter (Xia et al., 2015), expressed primarily in roots and stems, followed by lemma and palea. It was also found that potassium (K) levels were also affected on account of overexpression and knockouts of the gene. The property of non-specificity leads to reasonable suspicion that this gene could be a candidate for investigation for its role in Si or As transport and distribution.

Other than genes directly involved in Si transport, certain other genes that fulfill the role of regulation as well as the genes proposed to be upregulated under the Si amendment were found in the QTL regions in our study. Thirteen cyclin-like F-box domain-containing proteins were detected in our study, possibly linked to Si regulation. Twelve of them were found in a single QTL, *qSSi10*, while one was found in *qSSi2*. (**Table S5**). Cyclin-like F-box domains are protein-protein interaction mediators, the genes of which were upregulated upon external Si amendment in response to rice blast stress (Brunings et al., 2009). Also, twelve F-box domain proteins were found in the As QTL regions in a panel phenotyped for As concentration in flag leaf and dehulled grains (Frouin et al., 2019). This indicates the putative role of F-box domain proteins in Si and As regulation.

In addition, two genes encoding MYB transcription factors were detected in our study (*Os02g0271900* and *Os12g0586300*) (**Table S7**). MYB-related transcription factors have previously been shown to be involved in the regulation of expression of certain genes involved in heavy metal (Cd) and metalloid (As) detoxification and/or resistance/tolerance (Li et al., 2015; Roy, 2016; Wang et al., 2017; Xu et al., 2018). Wang et al. (2017) reported that *OsARM1*, an MYB transcription factor, was found to regulate the plant response to As stress. Also, Chen et al. (2021) proposed that an MYB factor gene, *OsMYBS1*, is responsible for Si-mediated As and Cd stress responses by regulating the expression of the transporter genes through GA or ABA signals (Lu et al., 2002; Chen et al., 2017; Chen et al., 2021). Additionally, a transcriptome study in wheat found that MYB factor genes formed a significant subset of the genes that were differentially expressed when the seedlings were treated with Si. These genes were involved in chaperones, folding catalysts, and protein processing in the endoplasmic reticulum (Hao et al., 2021). Hence, the candidate genes in our study require further study to comprehend the gene regulation mechanisms underlying Si and As dynamics.

Additionally, *qSSi4.1* contains the SNARE-interacting protein *Os04g0252400.* SNAREs were reported to be influencing the expression of the level of trafficking in PIPs (Plasma membrane-intrinsic proteins), which belong to the aquaporin protein family and are responsible for the transport of water and certain solutes, including silicic acid and arsenate (Tyrrell et al., 2007). Also, *qSSi3.2* bears another interesting gene that encodes a BTB-domain-containing gene, *Os03g0686050*. The *OsTAZ24* gene, which encodes a BTB-domain protein, was found to be responsible for promoting plant resistance to various heavy metals (Shalmani et al., 2021; Pinson et al., 2022). Hence, it has been considered a candidate gene in relation to responses to As or Si. Based on expression analysis, one gene coding for a non-protein-coding transcript was also found in relation to Si. The influence of non-coding RNAs on gene expression is an unchartered world that requires deeper understanding.

## Conclusions

Overall, the study has identified donors and QTLs for lower straw silica content in *O. nivara*, which would open avenues for manipulating silica content in rice for the judicious management of rice straw. The high phenotypic variation along with a weak population structure makes this panel very suitable for GWAS. GWAS identified 14 significant MTAs, of which six lie in previously reported QTL regions. Candidate genes, including the ABC transporter, Casparian strip proteins, MATE gene, F-box, and MYB-domain proteins, have been identified in the study. Future studies could be directed on multiple fronts, including the validation of candidate genes, deciphering the role of regulatory elements in Si transport and regulation, root architecture studies, comprehending the role of Si and the underlying mechanisms of Si in biotic and abiotic stress responses, and so on. The syntenic regions in the maize genome for Si QTLs could also be studied further. The candidate genes identified in the study will further enhance our understanding of the role of silica in rice and will develop a platform for targeted breeding for engineering silica content in rice.

## Data availability statement

The original contributions presented in the study are included in the article/**Supplementary Material**. Further inquiries can be directed to the corresponding author.

## Author contributions

DB conceived the idea, designed, and supervised the study. RSRG carried out phenotyping, analyzed genotypic and phenotypic data, carried out GWAS and other analyses, wrote the first draft of the manuscript. SS, RSG, and GM helped in phenotyping and interpretation. DB and RSRG edited and wrote final version of manuscript. All authors listed have made a substantial, direct, and intellectual contribution to the work and approved it for publication.
