# Supplementary material for: Genome wide association studies and candidate gene mining for understanding the genetic basis of straw silica content in a set of Oryza nivara (Sharma et Shastry) accessions
Source: Front Plant Sci. 2023 May 30;14:1174266. doi: 10.3389/fpls.2023.1174266 (PMC10266271; doi:10.3389/fpls.2023.1174266)
Supplement: Supplementary file 1 [file DataSheet_1.docx]

Supplementary Material

**Genome wide association studies and candidate gene mining for understanding genetic basis of straw silica content in a set of *Oryza nivara* accessions**

## Supplementary Figures


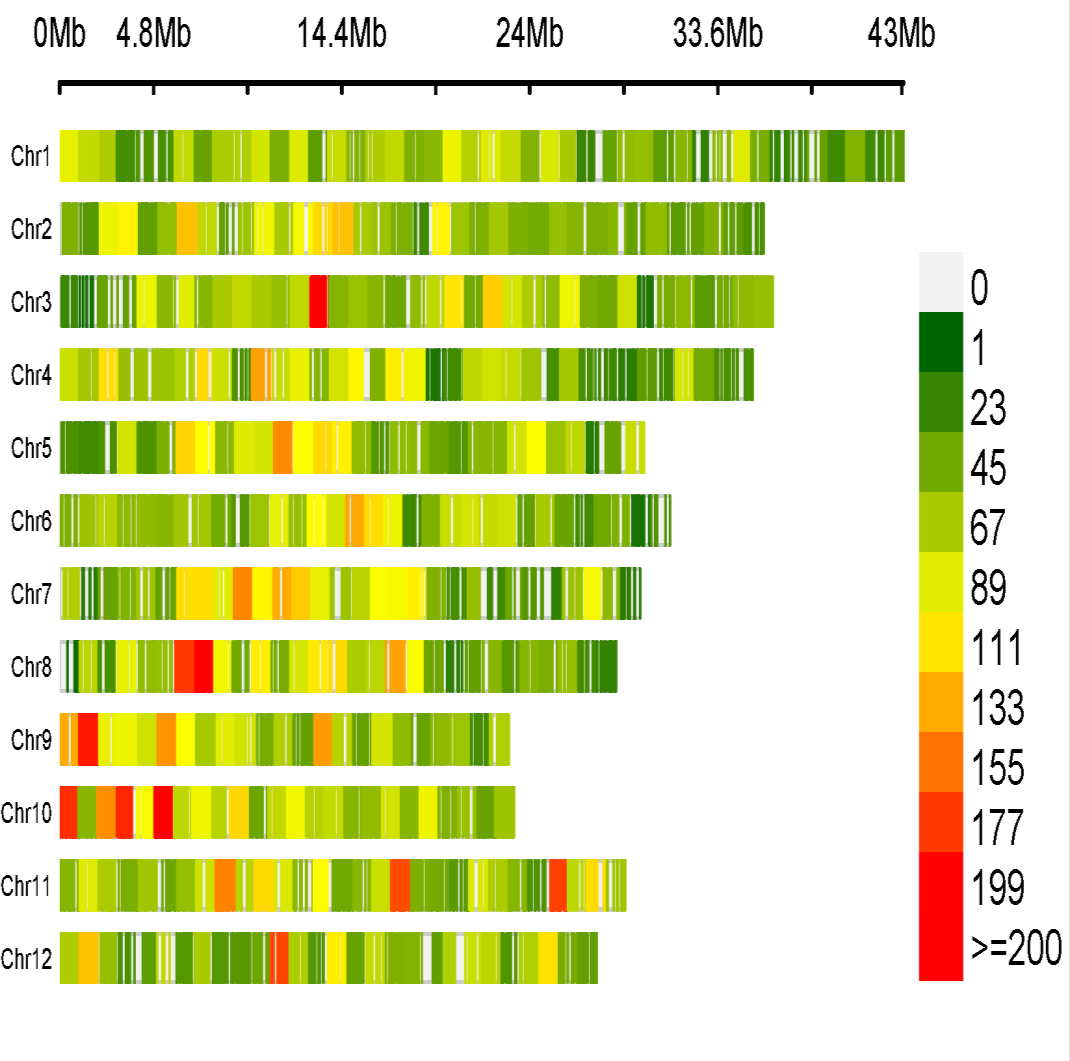


**Supplementary Figure 1.** SNP density plot depicting the SNP distribution along the 12 chromosomes. The different colour shades in the legend represent density of SNPs per Mb region along the genome


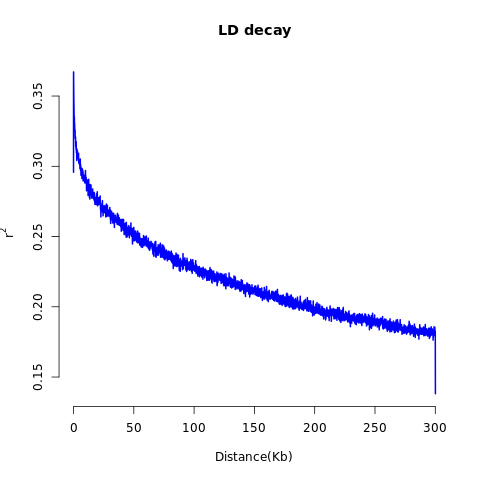


**Supplementary Figure 2.** Linkage disequilibrium (LD) decay across the entire genome in 258 *O. nivara* accessions


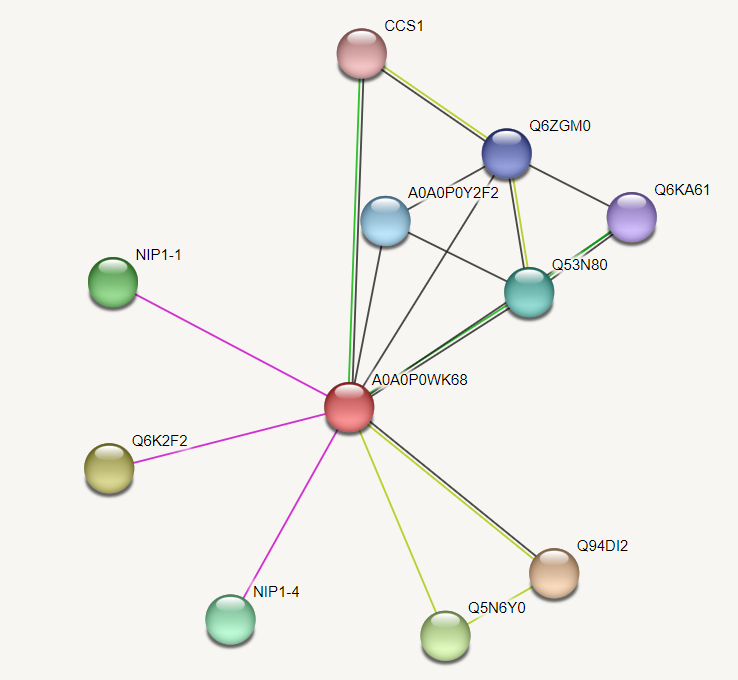


**Supplementary Figure 3.** Protein-protein interaction network of *Os05g0295200* (A0A0P0WK68, red circle in the centre). The nodes indicate the protein interactors
